# Cutaneous leishmaniasis mimicking psoriasis: A case report

**DOI:** 10.1002/ccr3.9299

**Published:** 2024-08-07

**Authors:** Saja Karaja, Mai Halloum, Shahed Karaja, Abdul hadi daher alhussen, Ayham Qatza, Sanaa Mansour, Alae Aldin Almasri

**Affiliations:** ^1^ Faculty of Medicine Hama University Hama Syria; ^2^ Ministry of Health Hama Syria

**Keywords:** case report, cutaneous, leishmaniasis, psoriasis

## Abstract

Scalp lesions associated with psoriasis and cutaneous leishmaniasis can be clinically indistinguishable, leading to misdiagnosis. Herein, we highlight a 70‐year‐old male initially misdiagnosed with psoriasis but subsequently confirmed to have cutaneous leishmaniasis. This emphasizes the importance of considering alternative diagnoses, especially in atypical presentations, to ensure accurate treatment.

## 1 INTRODUCTION

Psoriasis is a chronic, systemic immune‐mediated disorder characterized by the evolvement of pruritic, erythematous and scaly cutaneous plaques.^1^ Diagnosis predominantly depends on clinical assessment, with skin biopsies seldom needed.^2^ Chronic plaque psoriasis constitutes the most prevalent type, affecting 80%–90% of individuals.^2^ It typically manifests on the knees, elbows, scalp, and trunk.^1^ Scalp involvement is common in psoriasis patients, presenting with different phenotypes, and occasionally isolated scalp affliction is observed.^3^ Conversely, cutaneous leishmaniasis (CL) rarely affects the scalp and palms.^4^ CL is caused by the protozoan parasite *Leishmania*, vectored by the female sandfly.^5^ Worldwide, CL impacts approximately 12 million cases, with 2 million new occurrences identified each year.^5^ It predominates in around 100 endemic regions.^5^ Direct parasitological diagnosis confirms leishmaniasis infection.^5^ Following parasite inoculation, a papule emerges at the site, typically progressing to a nodule or plaque which tends to ulcerate.^6^ Available treatments are intralesional pentavalent antimonials, chemotherapy, cryotherapy, and thermotherapy.^6^ In this case report, a 70‐year‐old patient with a history of psoriasis presented with a crusted scalp lesion thought, initially, to be psoriatic and treated unsuccessfully with corticosteroids. Subsequent evaluation revealed CL, and it was successfully treated with antimonials, while it is unusual for leishmaniasis lesions to appear on the scalp.

## 2 CASE PRESENTATION

### 2.1 Case history

**FIGURE 1:**
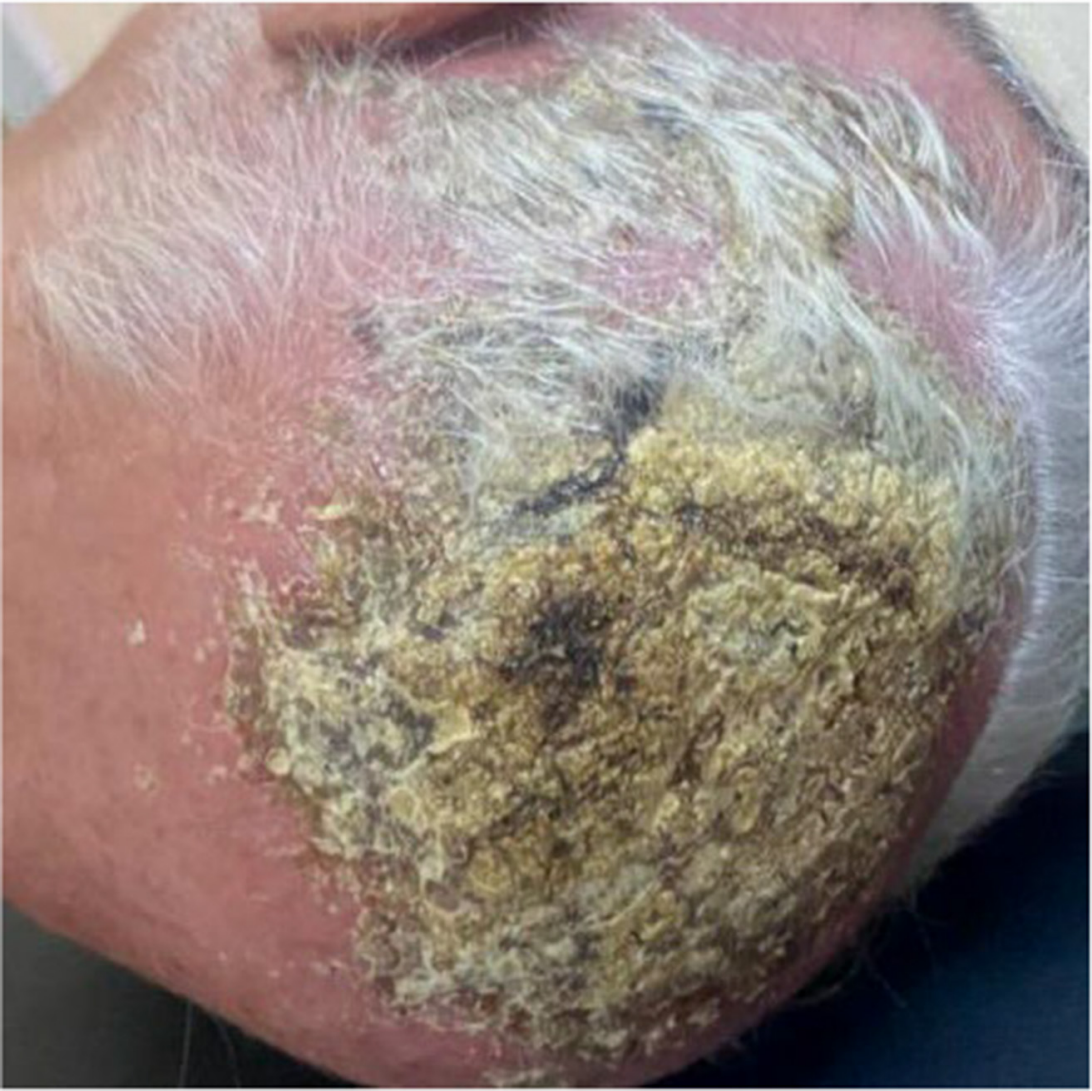
A presentation of a yellow‐tinted, crusted, erythematous plaque lesion on the scalp exhibiting signs of purulent exudation, accompanied by the presence of edema and erythema that appear to extend beyond the borders of the plaques.

A 70‐year‐old male, was previously diagnosed with psoriasis affecting the knees and the elbows, presented to the dermatology department complaining of an exuding lesion on the scalp. Clinical examination revealed a 10 × 10 cm yellowish, crusted, erythematous plaque lesion on the scalp with a purulent exudation, edema, and erythema extending beyond the plaques (Figure 1). In addition, there were no lesions or enlargements of the viscera or lymphadenopathy. The patient had received topical corticosteroids treatment for approximately 4 months before seeking care at our department as having a psoriatic lesion with no response.

### 2.2 Differential diagnosis, investigations, and treatment

**FIGURE 2:**
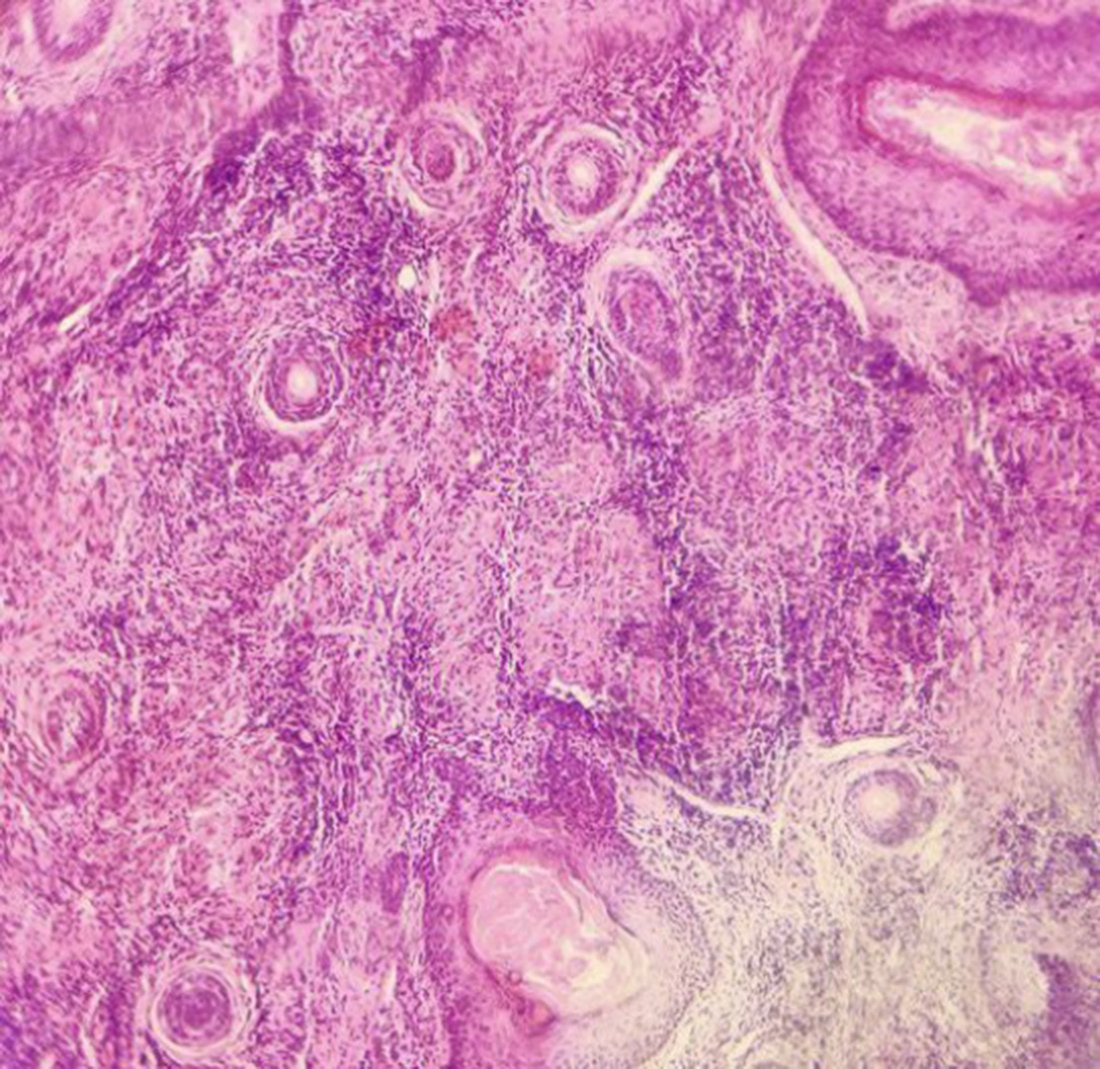
Histological findings: a presence of a granulomatous inflammatory process that is defined by the congregation of epithelioid cells, lymphocytes, plasma cells, and multifarious giant cells. No signs of malignancy or suggest cellular atypia.

Various differential diagnoses were considered including basal cell carcinoma (BCC), squamous cell carcinoma (SCC), scalp psoriasis, deep mycosis, and CL. Routine laboratory tests were within normal ranges. Histopathological findings displayed granulomatous inflammation characterized by epithelioid cells, lymphocytes, plasma cells, and some giant cells, with no indications of malignancy or cellular atypia (Figure 2), where the biopsied specimen was cut vertically. Due to unavailability of parasitological tests in our region, a diagnosis of CL was established based on clinical findings suggestive of leishmaniasis, histopathological findings of the biopsy, and a positive response to empirical therapy. Consistent with the protocol in these cases and after the cardiology consultation, the treatment included intramuscular injections of pentavalent antimonials (glucantime 7.5 mL) daily for 10 days, but the patient suffered from lower limb pain so the antimonial dose was reduced to 5 mL.

### 2.3 Outcome and follow‐up

**FIGURE 3:**
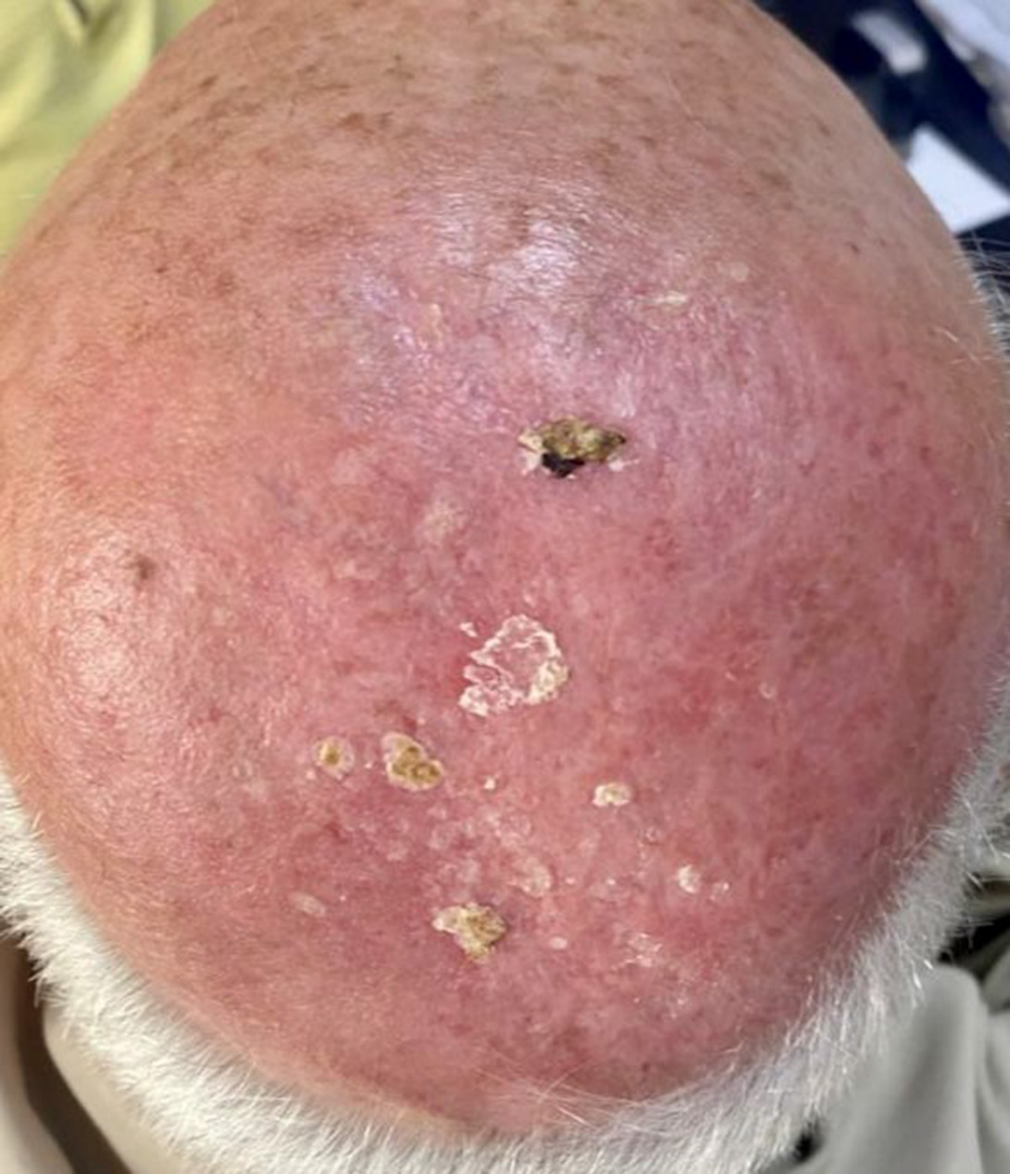
On Day 20 of administering pentavalent antimonials (glucantime 7.5 mL), there was a noted progressive diminution of the scalp lesion.

Subsequent follow‐up demonstrated a gradual regression of the scalp lesion, culminating in complete resolution after 20 days (Figure 3).

## 3 DISCUSSION

Psoriasis is acknowledged as a chronic inflammatory condition that impacts the skin, characterized by genetic and autoimmune features.^7^ The global incidence rate stands at approximately 2%, displaying variations across different geographic regions.^7^ Predominantly, it manifests on the extensor surfaces of the limbs, scalp, and trunk.^7^ Psoriasis primarily affects the skin, with occasional joint involvement in certain cases.^7^ Clinically, psoriasis presents as well‐defined, pruritic and erythematous plaques that can cover extensive areas of the skin with silvery scales.^7^ Symptoms of psoriasis include itching and bleeding, contributing to a significant burden for patients.^7^ Our patient, previously diagnosed with psoriasis on the knees and elbows, presented with a scalp lesion, a typical psoriatic site, leading to an initial misdiagnosis based on the common psoriatic manifestation of a yellowish, crusted, erythematous plaque lesion on the scalp, featuring purulent exudation. Among the various types of psoriasis, around 80%–90% of patients experience chronic plaque psoriasis, making it the most prevalent form.^2^ The patient exhibits a medical background of prior psoriatic involvement of the knees and elbows, thereby prompting consideration of a recurrent psoriatic lesion on the scalp, as psoriasis is associated with a chronic inflammatory condition. The initial diagnostic criterion for psoriasis is typically clinical evaluation.^2^ The patient received an initial misdiagnosis of psoriasis from another medical practitioner, relying solely on clinical observations and the patient's past history of psoriatic manifestations, without conducting supplementary diagnostic assessments. Psoriasis can often be misdiagnosed with conditions like atopic dermatitis, contact dermatitis, lichen planus, secondary syphilis and mycosis fungoides, with approximately 15% of cases remaining undetermined.^7^ Differential diagnoses of the scalp lesion in our patient involved BCC, SCC, scalp psoriasis, deep mycosis, and CL and they were sequentially ruled out. Patients with psoriasis commonly experience hypertension, type 2 diabetes, elevated hyperlipidemia, and coronary artery issues.^7^ Various risk factors linked to psoriasis include smoking and alcohol consumption, with additional associations to elevated cancer risks.^8^ Our patient experienced none. Various agents are under development for psoriasis therapy.^9^ Corticosteroids are recognized as the primary topical therapy for psoriasis treatment.^2^ Our patient was treated by topical corticosteroid when he was misdiagnosed with psoriasis. In cases where topical treatments prove ineffective, phototherapy emerges as a key choice for moderate to severe psoriasis.^2^ The Food and Drug Administration (FDA) in the United States has approved TNF‐α inhibitors as a primary therapy for psoriasis.^7^ For patients unresponsive to conventional systemic treatments, biological therapy is recommended as a potent alternative.^2^ In contrast, leishmaniasis, recognized as one of the neglected tropical diseases,^10^ ranks as the third most significant vector‐borne ailment globally.^10^ It predominantly impacts impoverished populations residing in regions surrounding the Mediterranean Basin, East Africa, the Americas, and Southeast Asia.^11^ Endemic in 98 nations, merely eight countries, including Syria, contribute to 90% of reported cases.^5^, ^11^ Considering the patient's Syrian origin, a region recognized for its endemicity concerning CL, the prudent examination of this affliction in correlation with the patient's lesion was deemed essential. In 2019 alone, the Syrian Arab Republic recorded 89,357 cases of leishmaniasis.^10^ In January 2020, approximately 6178 CL cases were reported.^10^ Recent conflicts in Syria triggered CL outbreaks due to healthcare disruptions and potential human‐to‐human transmission amid high‐density living conditions.^5^ Incidence figures in most regions are likely underestimated due to under‐recognition and non‐mandatory reporting.^5^ *Leishmania*, is an obligate intracellular parasite belonging to the order Kinetoplastida and the family Trypanosomatidae,^10^ infiltrates phagocytic host cells.^3^ Transmission predominantly occurs through blood‐feeding female sandflies.^10^ Optimal sandfly activity occurs during warm, calm nights with minimal wind.^10^ Leishmaniasis can manifest in three primary clinical forms: localized cutaneous leishmaniasis (LCL), muco‐cutaneous leishmaniasis (MCL) involving mucosal tissues, and visceral leishmaniasis (VL) affecting internal organs like the liver, spleen, and bone marrow. VL, akin to MCL, can be fatal, though less common.^5^ American tegumentary leishmaniasis represents a fourth syndrome caused by New World Leishmania species, encompassing CL and MCL presentations primarily, alongside rarer forms like diffuse and disseminated CL.^5^ *Leishmania* parasites are categorized into two predominant groups based on the European perspective: (1) Old World species prevalent in regions like the Mediterranean Basin, the Middle East, and the horn of Africa; and (2) New World species prevalent in Middle and South America. Old World species typically cause self‐restricting ulcers, contrasting with the potentially severe and even lethal outcomes attributed to New World species, particularly in MCL cases.^5^ LCL stands as the most prevalent form of leishmaniasis characterized by lesion persistence ranging from months to years. CL lesions often evolve from papules to nodular plaques to ulcerative lesions, with variable appearance and size alterations over time.^4^ Clinical examination for our patient revealed a 10 × 10 cm yellowish, crusted, erythematous plaque lesion on the scalp with a purulent exudation and an edema, and it is unusual for leishmaniasis papules and nodular plaques to present as a psoriasis crusted lesion. The singular clinical observation that facilitated the contemplation of leishmaniasis within our locale pertained to the presence of erythema surpassing the boundaries of the plaques. Uncommon sites, like the scalp and palms, can also be affected by CL.^4^ That is why the scalp lesion was lately diagnosed. Detection methods for diagnosing leishmaniasis include direct parasitological examination (microscopy, histopathology, and parasite culture) that has a high specificity, and indirect testing with serology and molecular diagnostics.^5^ Molecular diagnostics play a crucial role in determining *Leishmania* species, which plays a crucial role in CL management, but are often unavailable in resource‐limited regions.^5^ We depended on the histopathological findings that displayed a granulomatous inflammation characterized by epithelioid cells, lymphocytes, plasma cells and some giant cells, and due to unavailability of parasitological tests in our region, we were unable to detect the *Leishmania* species because it is unavailable in our region and the diagnosis of CL was established based on clinical findings suggestive of leishmaniasis and positive response to empirical therapy. CL is typically self‐limiting.^5^ In cases where species identification is lacking, treatment decisions rely on local medical expertise.^5^

Pentavalent antimonials (sodium stibogluconate, pentostam, meglumine antimoniate and glucantime) are the first‐line treatment for CL in many countries.^5^ Systemic therapy is advisable for patients with multiple lesions, facial involvement, or lesions unsuitable for topical treatment.^5^ There is currently no ideal vaccine or definitive treatment for leishmaniasis eradication.^11^ For local CL therapy, the World Health Organization (WHO) recommends injecting 1–3 mL of pentavalent antimony solution beneath the lesion until the skin blanches, repeated every 5–7 days for two to five treatments.^11^ Because of the large size of the lesion and the available treatments in our country, the treatment for our patient included intramuscular injections of pentavalent antimonials (glucantime 7.5 mL) daily for 10 days after cardiac consultation and normal medical tests, then the dose was reduced to 5 mL until the lesion ended with complete resolution after 20 days.

## 4 CONCLUSION

Establishing a definitive diagnosis of recurrent psoriasis based solely on a history of previous infection and clinical manifestations may prove inadequate. Notably, although CL seldom affects the scalp and palms, such atypical presentations are plausible. Therefore, in geographic areas endemic to CL, any cutaneous lesion resembling psoriasis should prompt thorough evaluation to consider CL as a plausible etiology.

## AUTHOR CONTRIBUTIONS

**Saja Karaja:** Writing – original draft; writing – review and editing. **Mai Halloum:** Writing – original draft. **Shahed Karaja:** Writing – original draft. **Abdul hadi daher alhussen:** Writing – original draft. **Ayham Qatza:** Writing – original draft. **Sanaa Mansour:** Data curation; writing – original draft. **Alae Aldin Almasri:** Supervision.

## FUNDING INFORMATION

There was no funding support for the case report.

## CONFLICT OF INTEREST STATEMENT

All authors declare that they have no conflicts of interest.

## ETHICS STATEMENT

Not applicable because all data belong to the authors of this article.

## CONSENT

Written informed consent was obtained from the patient to publish this report in accordance with the journal's patient consent policy.

## Data Availability

Data sharing not applicable to this article as no datasets were generated or analyzed during the current study.
